# Streptozotocin induced hyperglycemia in the axolotl

**DOI:** 10.1002/dvdy.70063

**Published:** 2025-07-23

**Authors:** Pernille Lajer Sørensen, Anita Dittrich, Henrik Lauridsen

**Affiliations:** ^1^ Comparative Medicine Lab, Department of Clinical Medicine Aarhus University Denmark

**Keywords:** *Ambystoma mexicanum*, β cell, diabetes, insulin, pancreas, regeneration

## Abstract

**Background:**

Diabetes is a group of diseases characterized by loss of β cell mass and/or function, resulting in hyperglycemia. With no established curative treatment, this has initiated research in β cell regeneration. Current animal models have either limited regenerative capacity (mice) or small size and evolutionary distance from humans (zebrafish). There is a need for new models to study endogenous regeneration pathways. This study proposes the axolotl salamander (*Ambystoma mexicanum*) as a model for studying the regeneration of β cells and aims to establish a protocol for STZ‐induced hyperglycemia to mimic a diabetic state.

**Results:**

In this pilot study, five streptozotocin (STZ) protocols were tested, and the most effective one was identified on the basis of glucose tolerance tests. Blood glucose levels were monitored to track both disease progression and remission. Histological examination of the pancreas and systemic effects of STZ treatment were also evaluated.

**Conclusion:**

Induction of a diabetes‐like state (hyperglycemia) in axolotls was possible with STZ, but variability among animals suggests the need for a higher degree of normalization or larger sample sizes. Histological regeneration was not observed, though blood glucose levels normalized over time. Some STZ‐treated animals developed edema, but its cause remains unknown.

## Key Findings

- The study demonstrates that a diabetes like state of hyperglycemia can be obtained in the axolotl by a fine tuned treatment with streptozotocin – a drug widely used in other diabetes models – with indications of recovery suggesting potential regeneration of pancreatic β‐cells.

## 1 BACKGROUND

Diabetes is a group of metabolic diseases, which are caused by the loss of β cell mass or function, resulting in hyperglycemia.^1^ This chronic hyperglycemic state can result in long‐term damage to organs such as the vascular system, heart, eyes, kidneys, and liver.^2^ Diabetes has traditionally been categorized into different types, with type 1 and type 2 being the most well‐known.^1^, ^2^ Broadly, type 1 diabetes results from cell‐mediated autoimmune destruction of β cells, leading to absolute insulin deficiency, while type 2 diabetes is characterized by insulin resistance and relative insulin deficiency.

According to the International Diabetes Federation (2021), 537 million people are estimated to suffer from diabetes, with numbers expected to reach 643 million people in 2030, making it one of the fastest‐growing global health emergencies today.^3^, ^4^ Thus, diabetes is an increasing global health care problem, and a solution is imminently needed.

Diabetes is diagnosed based mainly on blood glucose measurements. In the clinical setting, hyperglycemia is detected by measuring fasting blood glucose after at least 8 h of fasting, performing a glucose tolerance test by administering a known oral glucose dose and measuring blood glucose 2 h later, or by the A1C test, which measures glycated hemoglobin, reflecting average glycemic levels over the past 2–3 months.^1^

Untreated diabetes can have severe consequences due to the toxicity of elevated blood glucose; thus, exogenous insulin is the mainstay therapy in most cases.^5^ Glycemic control is maintained through subcutaneous insulin injections, requiring self‐monitoring of blood glucose.^2^ Importantly, incorrect insulin dosage can lead to hyperinsulinemia and hypoglycemia, which, in extreme cases, may be fatal.^2^

Curative strategies focus on restoring lost β cells to reestablish normal insulin secretion. Research in this area spans nearly a century, yet no ideal treatment exists.^6^ Investigated methods include cadaveric pancreas/islet transplantation, differentiation of pluripotent stem cells into β cells or pancreatic progenitors, β cell replication stimulation, reprogramming of non‐β cells, xenotransplantation, and transplantation of animal‐grown human pancreas.^7^ Each approach faces challenges, such as immune suppression requirements, scalability issues, invasive procedures, and identifying optimal targets for β cell regeneration. While significant progress has been made, each of these strategies faces major challenges, and further research is needed to develop a viable long‐term treatment for diabetes.

An emerging model that may offer insights into potential solutions lies in the remarkable regenerative capabilities of certain species, such as the axolotl. Unlike most mammals, the axolotl can regenerate complex structures,^8^, ^9^ including but not limited to the limbs, heart, liver, lungs, and spinal cord.^10^, ^11^, ^12^, ^13^, ^14^ This natural regenerative ability, particularly its potential for pancreatic tissue regeneration, makes the axolotl an intriguing candidate for studying β cell replacement. Given the increasing urgency to find a cure for diabetes, understanding how the axolotl achieves such regeneration could provide valuable insights into new therapeutic strategies for β cell restoration.

To understand how axolotls could play a role in this search for a cure, it is important to first consider the structure and function of the pancreas, an organ that plays a central role in both digestion and metabolism.

The pancreas is a long, slender organ connected to the digestive tract, with both exocrine and endocrine functions. The exocrine pancreas produces digestive enzymes, while the endocrine pancreas regulates metabolism through hormone secretion. In mammals, endocrine cells are clustered into islets of Langerhans, comprising five primary cell types producing distinct hormones, one of these being insulin‐secreting β cells.^7^ The secretion of insulin depends on circulating nutrients, especially glucose. In a healthy pancreas, increased plasma glucose triggers insulin release while suppressing glucagon secretion from α cells, and vice versa.

The development and histology of the pancreas are largely conserved across mammals, birds, reptiles, amphibians, and some fish.^15^ While the axolotl pancreas remains unstudied, investigations in Xenopus, a widely used amphibian model, have identified most cell types and a developmental process similar to mice and humans.^16^ Insulin expression has been identified throughout the entire pancreas with the endocrine cells arranged in clusters, as seen in other species.^16^ This comparability in pancreatic histology enhances the use of diverse animal models for studying pancreatic diseases, including diabetes.

The role of insulin in amphibians is less understood than in mammals, but studies on pancreatectomy in anurans have demonstrated hallmarks of diabetes, such as hyperglycemia, glucosuria, polyuria, increased ketone bodies, weight loss, and reduced glycogen stores.^17^ Notably, some toads showed pancreatic regeneration. In *Bufo arenarum*, an elevation in blood glucose and ketone levels occurred immediately after the operation but worsened over a period of 13 days (blood glucose and ketone levels were not measured afterward). This delayed hyperglycemic response was also seen in lizards (*Tupinambis teguixin*), in which the initial response was hypoglycemia, and hyperglycemia only occurred 8 weeks postoperatively.^17^ Urodeles are even less studied than anurans, but a study in *Taricha torosa* showed that pancreatectomy also resulted in an elevation in blood glucose levels.^17^ Moreover, studies in amphibians also showed that injections of insulin resulted in hypoglycemia, but the action of insulin was delayed and of prolonged duration, depending on the metabolic rate of the animals.^17^

The glucose tolerance test has been used in anuran to investigate glucose consumption, and a study showed that by intravenous (i.v.) injection of 3 mg/g body mass of glucose in male toads (*Bufo marinus*), glucose levels returned to fasting levels after 10 h.^17^ Other anuran studies reported normalization times between 8 and 24 h, demonstrating a much slower glucose metabolism than in humans.

In metabolic studies of amphibians, it is worth noting that amphibians are carnivores, and their macronutrient requirements are different from species such as rodents because they rely more on lipid/protein uptake than carbohydrates.^18^ In other carnivores, such as the domestic cat, protein has a more prominent role as a gluconeogenic substrate than carbohydrates.^19^

Research in animal models, such as mice and zebrafish, has identified three primary sources of new β cells: (1) proliferation of existing β cells, (2) neogenesis from stem or duct‐associated progenitor cells, and (3) transdifferentiation of other islet cells (e.g., α or δ cells).

In mammals, β cell proliferation is most active in youth but declines with age, though it can be reactivated under stress, such as pregnancy.^7^, ^20^, ^21^, ^22^ However, this capacity is limited in adults. Neogenesis, while observed in some studies, shows mixed results, with evidence of β cell formation from duct cells in zebrafish,^23^ but inconsistent findings in mammals.^24^, ^25^, ^26^, ^27^, ^28^ Transdifferentiation also plays a role, where α cells or δ cells can convert into β cells, a process that is especially pronounced in regenerative contexts in zebrafish^29^, ^30^, ^31^, ^32^, ^33^ and some mammals.^22^, ^34^

Overall, previous studies have identified several similarities, as well as notable differences, between regenerative diabetes models in mice, zebrafish, and amphibians (Figure S1). Therefore, understanding these pathways in regeneration‐positive models is essential for advancing β cell replacement therapies.

In amphibians, blood glucose levels are regulated by insulin secretion, as in humans, but the metabolic rate of glucose is lower than seen in mice and humans. While several amphibians have been shown to develop hyperglycemia, none have been shown to undergo intrinsic regeneration of β cells.

The axolotl salamander is a promising alternative to mice because it is highly regenerative compared to most other species (including other amphibians). While mice are the gold standard in diabetes research, particularly for studying β cell function due to their genetic similarity to humans, they lack the regenerative capabilities seen in axolotls. Mice do not naturally regenerate β cells to the extent that axolotls may, making the latter an interesting model for studying β cell regeneration in a way that mice cannot replicate.

Axolotls also offer advantages due to their larger size (up to 30 cm), enabling techniques such as serial blood sampling, advanced surgical procedures, and high‐resolution imaging, all of which are difficult to perform in smaller model organisms like zebrafish.^11^ Zebrafish are more well‐studied genetically, but both the axolotl genome and transcriptome have been sequenced, and several transgenic models have been developed.^35^, ^36^, ^37^, ^38^, ^39^ The axolotl represents a promising model for studying β cell regeneration following type 1 diabetes induction thanks to its capacity for tissue regeneration, accessibility, and applicability to a variety of experimental conditions. Understanding species like the axolotl that possess natural regenerative abilities is crucial for gaining insights into the limitations of human regenerative capacity and could eventually inform strategies to induce regeneration in humans. Here, we report the first study of successful STZ‐induced hyperglycemia in the axolotl, along with the subsequent functional restoration of β cells.

## 2 METHODS AND MATERIALS

### 2.1 Experimental design

#### 2.1.1 Pilot studies

A series of pilot studies were performed to optimize the STZ protocol for inducing diabetes in axolotls while minimizing mortality (see Figure S3 for information on animal size and number of individuals per group). A previously used zebrafish‐inspired protocol (0.35 mg/g body mass injected i.p. on days 0, 1, 2, 11, and 18)^40^ resulted in high mortality of axolotls.^41^ Instead, we tested several dosage regimes inspired by rodent studies (Figure S2).

Pilot 1 received 0.05 mg/g for five consecutive days; Pilot 2 received 0.05 mg/g on days 0, 2, 4, 12, and 19; Pilot 3 received a single 0.2 mg/g dose at day 0; Pilot 4 received 0.15 mg/g for five consecutive days; and Pilot 5 received 0.2 mg/g for five consecutive days. Fasting blood glucose level and glucose tolerance tests were used to determine the optimal protocol.

#### 2.1.2 Longitudinal glucose monitoring

Using the optimal STZ protocol from the pilot experiments, two groups were established for longitudinal glucose monitoring: one treated with STZ and one with sham treatment. The STZ‐treated group underwent glucose tolerance tests on days 22, 32, 42, 52, and 72, while the sham group was tested on days 22, 42, and 72. Both groups were terminated on day 84 for tissue harvest.

#### 2.1.3 Evaluation of disease progress, potential regeneration, and systemic effects

Following the longitudinal 84 days long glucose monitoring experiment in which a hyperglycemic response to STZ treatment was established and a potential restoration of β cell function was observed, two additional sets of STZ‐treated and sham‐treated groups were prepared for intermediate time point termination at day 22 (peak hyperglycemia) and day 35 (proposed initiation of potential β cell regeneration) (Figure S2). The diabetic state was thus evaluated at days 22, 35, and 84 following STZ treatment by measuring blood glucose levels and by histological examination of the pancreas (see Figure S2 for information on animal size and number of individuals per group). Potential whole‐body effects of STZ‐induced diabetes were characterized by measuring changes in body mass, the percentage of red blood cells, plasma osmolarity, and various plasma and urine parameters as described below.

### 2.2 Axolotl husbandry

Axolotls of mixed sexes and colors were acquired from a local breeder and housed in groups of 1–3 animals per cage. Animals were housed in containers (39 cm × 28 cm × 14 cm) with unchlorinated tap water and a water depth of 10 cm at 19–22°C and exposed to 12 h light–dark cycles. Water was changed 2–3 times/week, and animals were fed three food pellets 3 days/week (Axobalance adult Aquaterratec).

They were fasted during the acclimatization period to minimize weight fluctuations caused by food intake, ensuring a stable baseline weight for accurate STZ dosage during the treatment period. Throughout the treatment period, animals given STZ were kept under a fume hood to prevent contamination of the environment with highly toxic STZ. Water depth was reduced to 6–7 cm during injections to minimize the amount of STZ‐contaminated water. All procedures were approved by the Danish National Animal Experiments Inspectorate (protocol# 2020‐15‐0201‐00688) (Figure S3).

### 2.3 Anesthesia

Animals were anesthetized by full‐body submersion in 200 mg/L benzocaine (Sigma‐Aldrich) dissolved in 2–3 mL acetone, in 2 L water tanks for 20 min prior to STZ‐, EdU‐, and glucose injection and all blood collections, and for at least 30 min before euthanasia. Axolotls were kept in paper towels soaked in anesthetic water during procedures to maintain anesthesia. Axolotls absorb benzocaine transdermally and via the gills and regain consciousness within an hour after being returned to housing water.

### 2.4 STZ injection

Injections were performed under a fume hood with protective equipment due to the toxicity and risk to human health posed by STZ (double nitrile gloves and a back‐closure gown). STZ (EMD Millipore, cat. no. 572201) was stored at −20°C prior to use and was diluted in axolotl Ringer's solution (5750.8 mg/L NaCl, 130.7 mg/L KCl, 130.7 g/L CaCl_2_, and 174.3 mg/L NaHCO_3_) and mixed thoroughly; sham‐treated animals received Ringer's solution only. Dosages were adjusted to individual body mass, and the solution was administered within 30 min of preparation. Anesthetized animals were placed on wet blankets on a hard surface and given an i.p. injection using a BD Micro‐Fine 0.5 mL insulin syringe (30 G). After injection, animals were returned to their housing and monitored until normal gill movement resumed. Animals were fasted 24 h prior to each injection and 1 week before the first injection.

### 2.5 Glucose tolerance test

Animals were fasted for 3 days before each glucose tolerance test. Fasting blood glucose levels were measured, after which a 30% D(+)‐glucose solution (1.75 mg/g body mass; Sigma, cat. no. G8270) was injected into the jugular vein. Blood glucose was monitored using a handheld FreeStyle Precision Neo glucometer and test strips (Abbott). For non‐endpoint measurements, only a few drops of blood (~5–10 μL) were collected from a small puncture in the gill artery using a heparin‐coated needle to minimize blood loss and animal stress. Animals were returned to their tanks between sampling time points. In pilot experiments evaluating sampling safety, higher volumes (30–60 μL) were drawn during the test as described in the Results section. Unless otherwise noted, all blood glucose and ketone measurements involved minimal‐volume sampling.

In the pilot studies, measurements were taken at 0 h, 10 min, 5, 9, 14, and 24 h post‐injection. In the main study, measurements were taken at 0 h, 10 min, 14, and 24 h, and before euthanasia at 0 h, 10 min, and 9 h.

### 2.6 Blood ketone measurements

Blood ketone was measured before the glucose tolerance test using the same handheld glucometer with Abbott ketone strips, with a minimal blood volume (~5–10 μL) collected from the gill artery.

### 2.7 Tissue sample harvest

For biochemical analyses, blood was collected terminally either from the gill arteries or directly from the heart immediately after euthanasia. A heparin‐coated 0.5 mL insulin syringe was used initially to prevent clotting, followed by an EDTA‐coated syringe for additional collection. The full volume of blood was drawn whenever possible, depending on the size of the animal. Samples were processed by centrifugation at 6000 rpm for 5 min to separate plasma, which was transferred to a pre‐weighed tube. Tube weights were used to calculate red blood cell percentage, after which samples were snap‐frozen in liquid nitrogen and stored at −80°C.

The abdominal cavity was opened to expose the bladder, and urine was collected by carefully inserting a syringe while extending the bladder with tweezers; the urine was then transferred to a tube and snap‐frozen in liquid nitrogen.

**FIGURE 1:**
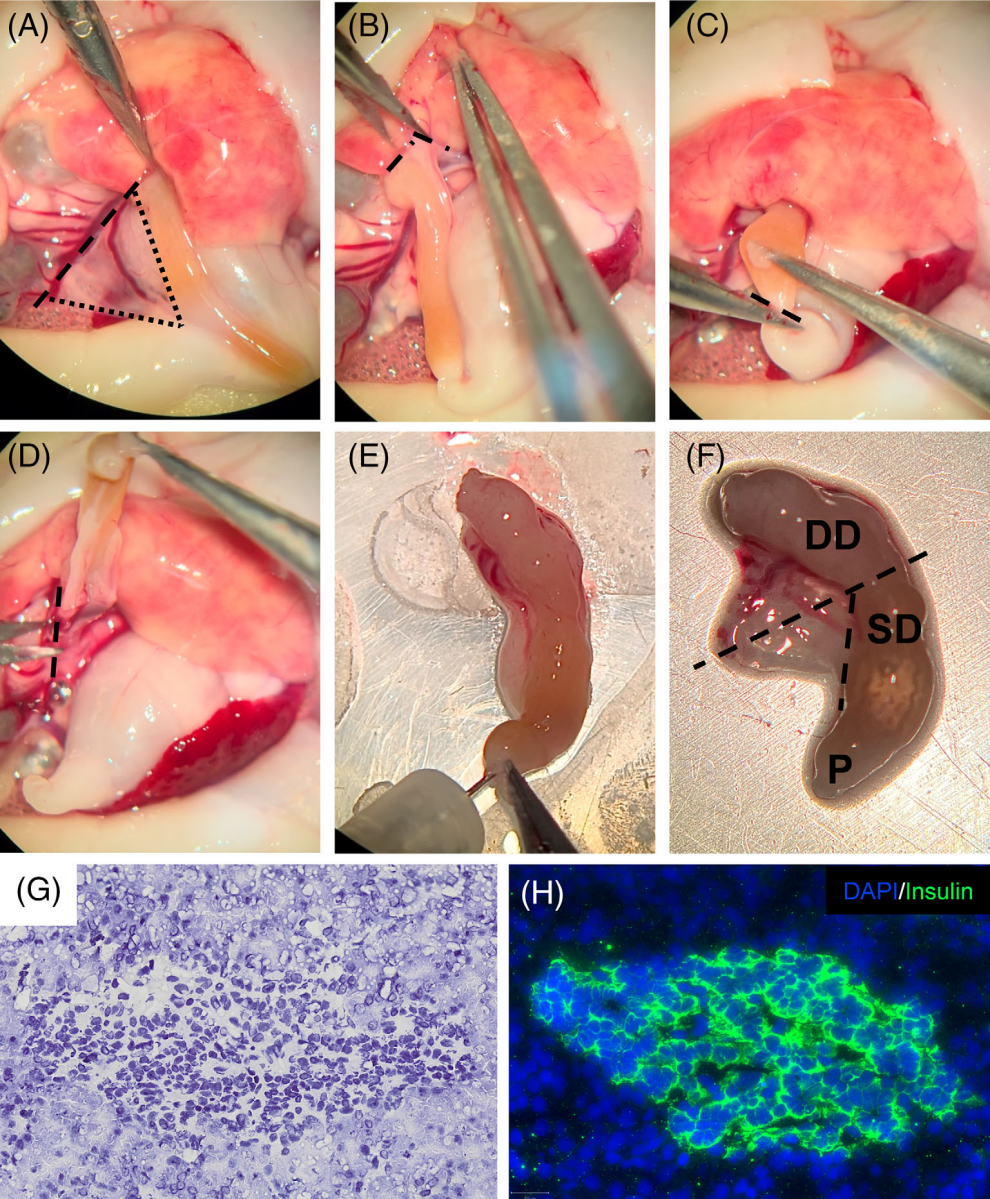
The axolotl pancreas. The dissection method of the pancreas. (A) The triangular pancreas, in which dotted lines frame the organ and the striped line shows the incision from connective tissue. (B) Pancreas is separated from the liver lobe. (C) The duodenum is cut near the pylorus. (D) Connective tissue is separated from the pancreas. (E) The duodenum is rinsed with amphibian Ringer's solution. (F) The pancreas is connected to a superior duodenal (SD) part near the pylorus (P) and a descending part (DD). Striped lines indicate the incision points, in which the part connected to SD is transferred to a tube (for other analysis), and the part connected to DD is transferred to a mold for histological analysis. (G) A β cell islet surrounded by exocrine pancreas in H&E‐stained tissue. (H) The same islet stained positive for insulin (green) and with nuclei (blue) staining.

The pancreas was removed by first separating the liver lobe from the pancreas, then severing the duodenum from the stomach, and severing pancreas tissue from connecting tissue (Figure 1). The pancreas was cleaned by rinsing with amphibian Ringer's solution through the duodenal opening and by removing blood clots. It was then cut at the midpoint: the half attached to the duodenum closest to the stomach was embedded in OCT and snap‐frozen in liquid nitrogen.

### 2.8 Osmolarity

Osmolarity was measured on blood plasma samples from swollen animals using an osmometer (Advanced Instruments, Inc., Model 3320).

### 2.9 Histology and immunostaining

Frozen pancreas tissue embedded in OCT was sectioned serially with a thickness of 7 μm, and neighboring sections were used for the different stains. Sections were stored at −80°C.

### 2.10 Hematoxylin staining

Sections equilibrated to room temperature (RT) for 5–7 min were fixed in methanol for 1 min, washed in demineralized water, and immersed in RT hematoxylin (Sigma‐Aldrich, cat. no. GHS316) for 1 min. After washing and drying, slides were mounted with Eukitt Quick‐hardening medium (Sigma‐Aldrich, cat. no. 03989) and coverslipped.

### 2.11 Staining of insulin‐positive cells in the pancreas

Sections were blocked in PBS with 3% BSA and 5% normal goat serum for 3 h at RT with gentle shaking. The primary insulin/proinsulin monoclonal antibody (ThermoFisher Scientific, cat. no. MA1‐83256) diluted 1:75 in 50% blocking buffer in PBS was applied overnight at 4°C in a humidity chamber. After 5 × 5 min PBS washes, a goat anti‐mouse IgG1 Alexa Fluor 488 secondary antibody (ThermoFisher Scientific, cat. no. A‐21121) diluted 1:500 in 50% blocking buffer was incubated for 3 h at RT in darkness. Following five additional PBS washes and air‐drying, DAPI Diamond mounting medium (ThermoFisher Scientific, cat. no. P36966) was applied. Slides were cured at RT for ≥24 h, sealed, and stored at 4°C in darkness until imaging.

To validate the specificity of the insulin/proinsulin antibody, standard negative control experiments were performed. These included omission of the primary antibody, omission of the secondary antibody, isotype control (IgG1), and staining of liver tissue, which does not contain insulin‐producing β‐cells. In all cases, no specific staining was observed.

### 2.12 Staining of proliferating cells in the pancreas

Sections were stained for proliferating cells using a Click‐iT EdU Imaging Kit (Invitrogen, cat No. C10637), in which 5‐ethynyl‐2′‐deoxyuridine (EdU) is incorporated into DNA during active DNA synthesis.

#### 2.12.1 EdU injection

Animals received an i.p. injection of EdU (0.035 mg/g body mass, dissolved in amphibian Ringer's solution) 24 h before euthanasia. The EdU amount was adjusted to body weight measured 1 week prior to the experiment to avoid inappropriate dosages due to edema development. Post‐injection, animals were kept on wet towels on ice for 1 h before returning to normal housing.

#### 2.12.2 Pancreas section processing

Sections were fixed in 4% PFA for 15 min, washed twice for 5 min in 3% BSA in PBS and twice for 5 min in 70% PBS, then permeabilized in 0.5% Triton X‐100 in PBS for 20 min. For EdU detection, prepared per manufacturer's protocol. Sections were counterstained with Hoechst 33342 solution (1:2000 in PBS) and mounted with ProLong Diamond Antifade Mountant (ThermoFisher Scientific, cat. no. P36965). Sections were kept in darkness at RT for 24 h, then stored at 4°C until imaging.

### 2.13 Imaging and analysis

Hematoxylin slides were captured at 20× magnification, and immunofluorescence slides at 40× magnification. Insulin‐ and EdU‐positive cells were quantified using QuPath v0.3.2, applying the same parameters such as sigma and threshold across samples. The pancreas was defined as the region of interest (guided by neighboring hematoxylin sections). Total cell counts were determined from the DAPI channel (using the Cell Detection tool). EdU‐positive nuclei were identified in the FITC channel, while insulin‐positive cells were classified by measuring FITC mean intensity after detecting DAPI‐positive cells. For each animal, four sections were analyzed, and the mean percentage of positive cells was calculated for statistical analysis.

### 2.14 Measurements of blood parameters with microplate assays

Albumin was measured from 20 μL plasma per animal by following the protocol of QuantiChrom BCP Albumin Assay Kit (BioAssay Systems, cat No. DIAP‐250). Alkaline phosphatase activity and creatinine were measured from 10 μL plasma using the QuantiChrom Alkaline Phosphatase (cat. No. DALP‐250) and Creatinine Assay Kits (cat. No. DICT‐500), respectively, with plasma diluted in distilled water to a total volume of 30 μL.

### 2.15 Glucose measurements of urine samples

Urine was initially measured for glucose using a FreeStyle Precision Neo glucometer before snap‐freezing in liquid nitrogen. Glucose was further quantified using a glucose assay kit II (Abnova, cat. no. KA0832) according to the protocol. Before assaying, urine samples were deproteinized with a Deproteinizing Sample Preparation Kit–TCA (Abcam, cat. no. ab204708) according to the protocol.

### 2.16 Protein measurement of urine samples

Using Pierce Microplate BCA Protein Assay Kit (Thermo Scientific, cat. no. 23252), protein was quantified in urine samples. The pH of samples from two animals per group was measured (pH 6–7.5) to ensure protocol compatibility.

### 2.17 Statistical analysis

All analyses were performed using the Real Statistics Resource Pack for Excel. Data are presented as mean ± standard deviation (SD), with *p* < .05 considered significant. Blood glucose levels at individual time points were compared between STZ‐treated and sham groups using unpaired two‐tailed Student's *t*‐tests with Bonferroni correction for multiple comparisons. Repeated glucose measures within the same animals were analyzed by one‐way repeated measures ANOVA with Tukey's HSD post hoc.

Glucose tolerance across STZ groups euthanized at days 22, 35, and 84 was assessed via two‐way repeated‐measures ANOVA, including Group (between‐subjects) and Time (within‐subjects) factors, with animal as a random effect. Post hoc comparisons used estimated marginal means with Tukey/Kramer adjustment. Sphericity was checked with Mauchly's test.

Blood (albumin, creatinine, alkaline phosphatase, red blood cells%, and osmolarity) and urine (glucose and protein) parameters were compared between STZ and control groups using two‐tailed Student's *t*‐tests, and among STZ groups using one‐way ANOVA with Tukey–Kramer post hoc. If sample sizes were too low to meet ANOVA assumptions, a Kruskal–Wallis test with Dunn's post‐hoc correction was used instead.

## 3 RESULTS

### 3.1 Characterization of the pancreas in the axolotl

To assess STZ's diabetogenic effects, we first characterized the axolotl pancreas, which is a triangular organ connected directly to the duodenum and liver (Figure 1). Exocrine and endocrine regions of the pancreas could be identified on hematoxylin‐stained sections (Figure 1), with endocrine cells forming islet‐like structures. Endocrine cells have a dark, well‐rounded nucleus and light cytoplasm, whereas exocrine cells have a less defined nucleus and light purple cytoplasm. β cells were identified by insulin staining, showing insulin accumulation in both the cytoplasm and nucleus (Figure 1).

### 3.2 Optimizing the glucose tolerance test

**FIGURE 2:**
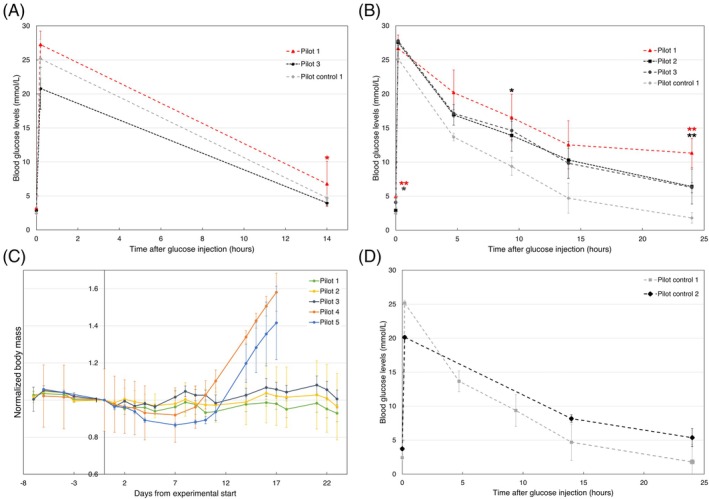
Pilot study results. (A, B) Blood glucose levels in pilot groups 1, 2, and 3 (each *n* = 3) on days 10 and 22, assessed by glucose tolerance tests and compared to Pilot Control 1 (*n* = 3). (C) Higher STZ doses (≥0.1 mg/g) led to increased body mass and required early euthanasia. (D) Glucose tolerance tests in healthy controls (Pilot Control 1 and 2, each *n* = 3) confirmed normal glucose levels over 24 h. Large blood collections (30–40 μL) did not significantly impact glucose levels in Pilot Control 2. *p < .05, **p < .01, ***p < .001, and ****p < .0001 (two‐tailed *t*‐tests).

The glucose tolerance test is used as a diagnostic tool in the clinic, but also in diabetes research to investigate disease in animal models. We have adjusted it for use in the axolotl (Figure 2), as the axolotl, like other amphibians,^17^ has a lower metabolic rate.

Intravenous glucose injection of 1.75 mg/g body mass resulted in a massive increase in blood glucose levels (approx. 22–27 mmol/L after 10 min), and approx. 60% of glucose is consumed after 14 h (Figure 2D). Blood glucose was measured at 0 h, 10 min, 5, 9, 14, and 24 h after glucose injection. Blood glucose returned to fasting levels after 24 h in healthy axolotls.

For axolotls to be a viable model in diabetes studies, the ability to perform serial blood sampling is essential. To determine whether larger blood collections affect glucose levels during the glucose tolerance test, a group of healthy axolotls (*n* = 6) underwent the optimized test, with 60 μL of blood collected at 0, 5, and 9 h. However, 4 out of 6 animals experienced complications recovering from anesthesia, and the experiment was terminated early. Notably, animals already showed signs of poor recovery following the 5 h collection, suggesting that the complications were primarily due to excessive blood loss rather than the frequency of sampling or anesthetic exposure. This interpretation is supported by a subsequent pilot study in which axolotls were sampled at similar time points (0, 5, and 10 h) but with lower blood volumes, and no adverse effects were observed.

To further confirm the tolerability of smaller blood volumes, a second control group (Pilot control 2, *n* = 3) was sampled with 30–40 μL collected at 0, 14, and 24 h. This group showed no complications, and blood glucose levels normalized by 24 h (5.4 ± 1.8 mmol/L at 24 h vs. 3.7 ± 0.7 mmol/L at fasting). These findings indicate that small‐volume serial sampling is safe in axolotls during the glucose tolerance test, while excessive cumulative blood loss should be avoided to minimize harm to the animals.

### 3.3 Finding the optimal STZ treatment protocol to induce diabetes in the axolotl

Ten days after the last STZ injection, blood glucose levels were not significantly higher at baseline for either Pilot 1 or Pilot 3 (two‐tailed *t*‐test: *p* = .31 and *p* = .57, respectively), but Pilot 1 displayed a significantly higher blood glucose level 14 h after glucose injection (two‐tailed *t*‐test: *p* = .040), whereas Pilot 3 did not differ from Pilot control 1 after 14 h (two‐tailed t‐test: *p* = .37) (Figure 2A).

On day 22, Pilot 1 and Pilot 3 showed significantly higher fasting blood glucose compared to controls (4.9 ± 0.2 mmol/L (two‐tailed *t*‐test: *p* = .0030) and 4.1 ± 0.002 mmol/L (two‐tailed *t*‐test: *p* = .012), respectively) compared to control (2.4 ± 0.4 mmol/L) (Figure 2B). After 5, 9, 14, and 24 h, all STZ‐treated groups had higher blood glucose levels compared to controls, with Pilot 1 showing the most severe hyperglycemic response, though not statistically significant. After 24 h, Pilot 1 had blood glucose levels of 11.3 ± 2.2 mmol/L (two‐tailed *t*‐test: *p* = .0093), Pilot 2 had 6.4 ± 2.6 mmol/L (two‐tailed *t*‐test: *p* = .21), and Pilot 3 had 6.3 ± 0.8 mmol/L (two‐tailed *t*‐test: *p* = .0034).

To further evaluate the optimal protocol without systemic toxicity, two additional groups (Pilot 4 and Pilot 5) were treated with 0.15 and 0.2 mg/g STZ, respectively, over five consecutive days. However, these groups experienced excessive weight gain due to edema (57.2 ± 15.1% for Pilot 4 and 44.0 ± 19.8% for Pilot 5), leading to premature termination of the study (Figure 2C).

Ultimately, the Pilot 1 protocol (0.05 mg/g STZ for five consecutive days) proved to be the most effective, inducing severe hyperglycemia without significant systemic toxicity by day 22.

### 3.4 A new STZ group was hyperglycemic on day 22 but showed signs of edema

Using Pilot 1's treatment regiment, we wanted to investigate if animals remained hyperglycemic after day 22, or if blood glucose levels normalized, indicating some degree of restoration of β cell activity, possibly through β cell regeneration.

For this, two groups of animals were included in a longitudinal experiment, one treated with STZ (*n* = 9) and the other receiving a sham treatment (*n* = 6). Weight, length, and colors did not differ significantly between groups (Figure S3). The STZ group was subjected to the glucose tolerance test on days 22, 32, 42, 52, and 72, whereas the sham group was subjected to the test on days 22, 42, and 72. To minimize blood loss during the glucose tolerance test, two measurements were excluded from the test, and blood glucose was measured 0 h, 10 min, 14 h, and 24 h after glucose injection.

By day 22, 3 out of 9 STZ‐treated animals exhibited swelling, and one was excluded due to a 89.2% weight increase. Glucose injections were weight‐adjusted, assuming even distribution despite swelling.

**FIGURE 3:**
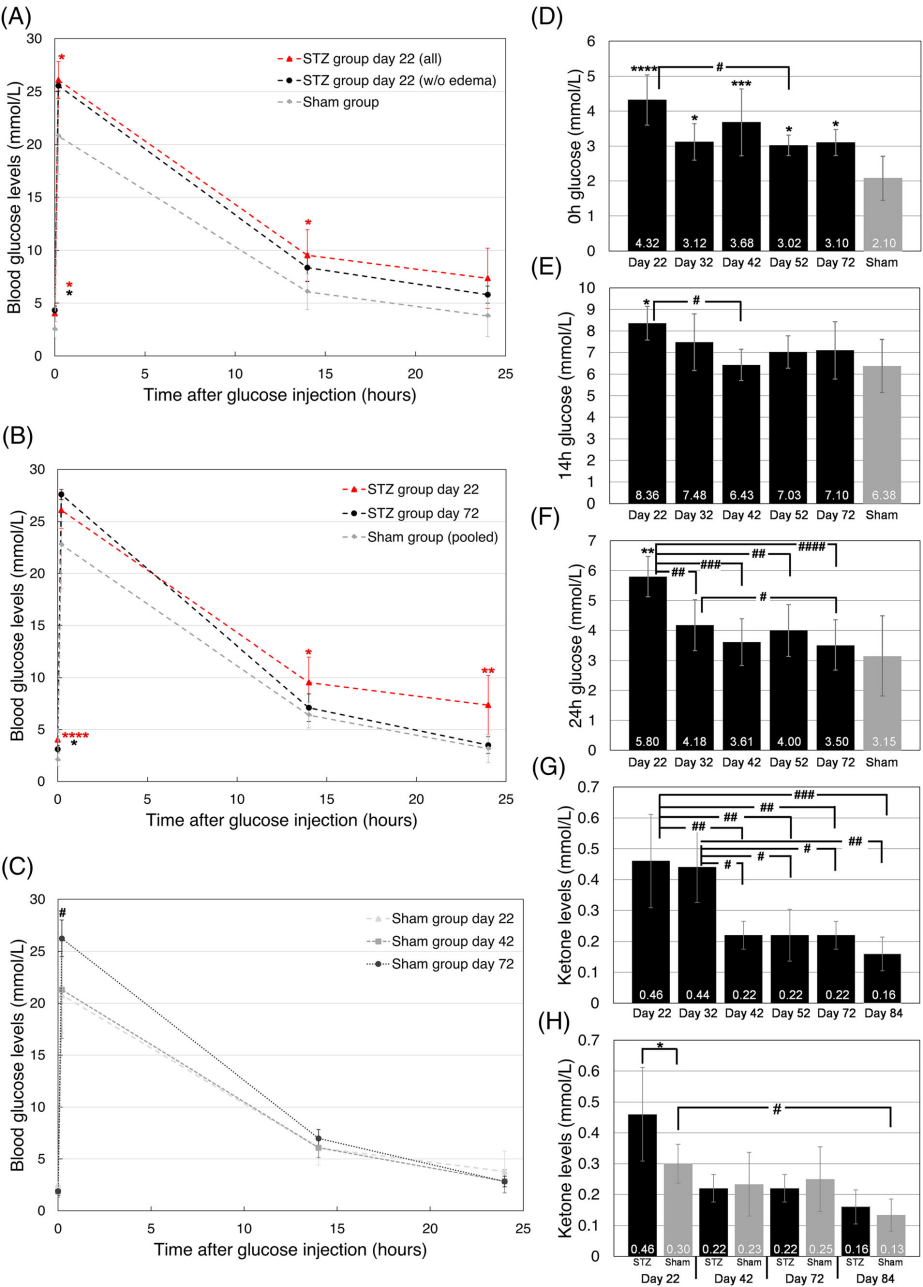
Longitudinal glucose and ketone monitoring in STZ‐treated axolotls. Blood glucose was assessed using glucose tolerance tests. (A) STZ‐treated animals, both with (*n* = 8) and without (w/o) edema (*n* = 5), were hyperglycemic on day 22. (B) Glucose levels normalized by day 72. (C) Sham‐treated animals (*n* = 6) maintained stable glucose levels across all time points, with 10 min readings not reflecting true glucose consumption. Glucose levels were compared at fasting (D), 14 h (E), and 24 h (F) post‐injection on days 22, 32, 42, 52, and 72. Ketone levels were measured on days 22, 42, 72, and 84, comparing within the STZ group (G) and between treatment groups (H). Significance levels are indicated as follows: *p < .05, **p < .01, ***p < .001, and ****p < .0001 (two‐tailed *t*‐tests); # *p* < .05, ## *p* < .01, ### *p* < .001, and #### *p* < .0001 (one‐way repeated measures ANOVA with Tukey's HSD post‐hoc test).

Blood glucose levels were significantly higher in the STZ group compared to the sham group, even after excluding swollen animals (Figure 3A). Fasting blood glucose was elevated in the STZ group (4.0 ± 0.7 mmol/L, two‐tailed *t*‐test: *p* = .011; excluding swollen: 4.3 ± 0.6 mmol/L, *p* = .011) compared to the sham group (2.5 ± 0.7 mmol/L). Post‐injection, glucose levels remained significantly higher in the STZ group at 10 min (26.1 ± 1.7 mmol/L, two‐tailed *t*‐test: *p* = .013) and 14 h (9.5 ± 2.4 mmol/L, two‐tailed *t*‐test: *p* = .047), but not at 24 h (7.5 ± 2.9 mmol/L, two‐tailed *t*‐test: *p* = .089), versus the control (10 min: 20.8 mmol/L, 14 h: 6.1 ± 1.7 mmol/L, 24 h: 3.8 ± 2.0 mmol/L) (Figure 3A).

When swollen animals were excluded from analysis, the differences in blood glucose levels between groups were no longer statistically significant, likely due to reduced sample size (Figure 3A). However, because sham‐treated animals did not differ significantly across time points, their data were pooled to increase statistical power. With this adjustment, the STZ‐treated group (excluding swollen animals) showed significantly elevated glucose levels at all measured time points (Figure 3B).

### 3.5 Blood glucose levels normalized over the span of 72 days

Blood glucose levels were monitored on days 22, 32, 42, 52, and 72 in the STZ group and on days 22, 42, and 72 in the sham group using the glucose tolerance test. Four animals from the STZ‐treated group were excluded due to swelling.

Blood glucose levels in the STZ group peaked at day 22 and gradually normalized by day 72, approaching levels seen in the sham group (Figure 3B). A one‐way repeated measures ANOVA with Tukey HSD post hoc test indicated significant differences in fasting blood glucose across days (*p* = .025), particularly between day 22 and 52 (*p* = .038) (Figure 3D).

Blood glucose levels measured 14 h after glucose injection also showed significant differences over time (*p* = .021) (Figure 3E), especially between day 22 and day 42 (*p* = .012). After 24 h, glucose levels differed significantly between test days (*p* = 6.67 × 10^−5^), particularly between day 22 and day 32 (*p* = .0032), day 42 (*p* = .00015), day 52 (*p* = .0012), and day 72 (*p* = 8.60 × 10^−5^) (Figure 3F).

The sham group had stable blood glucose levels at all but one time point (Figure 3C). At the 10 min measurement, blood glucose differed significantly between the measurement days (one‐way repeated measures ANOVA with Tukey HSD: *p* = .012), between day 72 and earlier time points (*p* = .019 on day 22, *p* = .0032 on day 42). These variations did not impact glucose consumption rates the remainder of the test, suggesting initial glucose distribution differences in the body tissues rather than true metabolic changes. Thus, blood glucose levels at each time point were pooled with those from the same time point on other test days for comparison with the blood glucose levels in the STZ group.

Comparing blood glucose levels of the STZ group to the sham group (each time point pooled for all measurement days), fasting blood glucose remained significantly higher in the STZ group at all time points (two‐tailed *t*‐test: *p* = 3.9 × 10^−6^ at day 22, *p* = .012 at day 32, *p* = .00083 at day 42, *p* = .018 at day 52, and *p* = .011 at day 72) (Figure 3D). However, fasting blood glucose decreased from 4.3 ± 0.7 mmol/L at day 22 to 3.1 ± 0.4 mmol/L at day 72, approaching control levels (2.1 ± 0.6 mmol/L). At 14 and 24 h post‐injection, glucose levels in the STZ group were significantly higher than the sham group only on day 22 (two‐tailed *t*‐test—14 h: 8.4 ± 0.8 mmol/L vs. 6.4 ± 1.2 mmol/L, *p* = .011; 24 h: 5.8 ± 0.7 mmol/L vs. 3.2 ± 1.3 mmol/L, *p* = .0015) (Figure 3E,F).

These results confirm that the STZ group experienced peak hyperglycemia on day 22, with blood glucose levels normalizing during the experiment.

### 3.6 Blood ketone levels were elevated on day 22 and decreased over time

Since ketone bodies serve as an alternative metabolic fuel in severe diabetes and have been observed in amphibians post‐pancreatectomy,^17^ blood ketone levels were measured before each glucose tolerance test.

In the STZ group, blood ketone levels significantly declined from 0.46 ± 0.02 mmol/L on day 22 to 0.16 ± 0.003 mmol/L on day 84 (Figure 3G). One‐way ANOVA with Tukey's HSD post‐hoc test confirmed a significant reduction over time (*p* = .00012), with high levels on days 22 and 32.

However, the sham group also exhibited a significant decline in blood ketone levels over time (*p* = .030), with significantly lower levels on day 84 compared to day 22. This suggests that factors beyond STZ treatment contributed to the overall decrease by day 84. However, the STZ group had significantly higher blood ketone levels than the sham group on day 22 (two‐tailed *t*‐test: *p* = .042) (Figure 3H), indicating that STZ treatment initially induced increased ketone production.

These findings suggest that while STZ treatment led to early hyperketonemia, ketone levels normalized over time, potentially due to β cell regeneration or other influencing factors.

### 3.7 STZ treatment did not induce hyperglycemia in new STZ‐treated animals terminated at intermediate time points

Since STZ‐treated animals in the first 84 days long longitudinal experiment were hyperglycemic on day 22, with blood glucose levels decreasing between days 22 and 42, two new sets of STZ‐treated and sham‐treated animals were prepared and euthanized on day 22 (peak hyperglycemia) and day 35 (potential start of regeneration), respectively. Since the batch of axolotls used for the pilot experiments and the first longitudinal experiment was exhausted, the animals in the intermediate time points groups consisted of animals from mixed batches. Despite internal and external differences in weight, length, and age in the animals euthanized on days 22 and 35 (Figure S3), all animals were analyzed under the assumption that the STZ protocol accounted for body mass differences. At euthanasia, all animals underwent a glucose tolerance test and were sacrificed 9 h post‐injection, with blood glucose measured at 0 h, 10 min, and 9 h.

**FIGURE 4:**
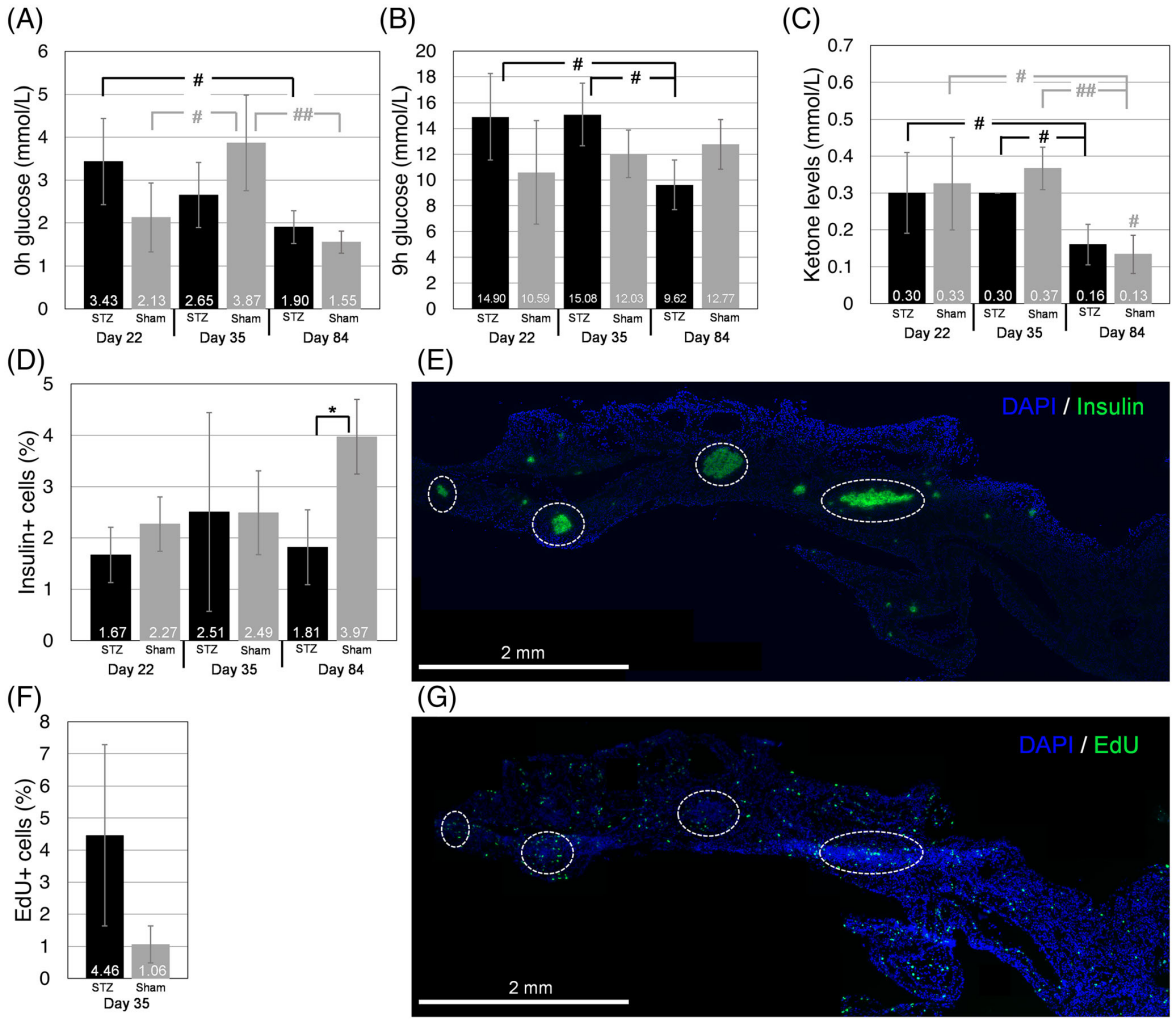
Disease progression in streptozotocin (STZ)‐treated axolotls. Glucose tolerance tests were performed on days 22, 35, and 84 in both STZ‐treated (day 22: *n* = 6; day 35: *n* = 4; day 84: *n* = 5) and sham‐treated animals (day 22: *n* = 4; day 35: *n* = 3; day 84: *n* = 6). Blood glucose was measured at fasting (A) and 9‐h post‐injection (B), while ketone levels were assessed at fasting levels (C). After euthanasia, pancreatic tissue was analyzed for insulin‐positive (+) cell percentage (D) and visualized via immunostaining (E, green). Sample sizes for insulin staining were: STZ‐treated: day 22: *n* = 4; day 35: *n* = 3; day 84: *n* = 3; sham‐treated: *n* = 3 for all time points. Proliferating (EdU+) cells were quantified (F) and localized in pancreatic tissue (G, green) in animals euthanized on day 35 (STZ: *n* = 4; sham: *n* = 3). DAPI staining was used to identify nuclei, and white circles indicate β cell islets. *p < .05, **p < .01, ***p < .001, and ****p < .0001 (two‐tailed *t*‐tests); # *p* < .05, ## *p* < .01, ### *p* < .001, and #### *p* < .0001 (ANOVA with Tukey's HSD/Kramer post‐hoc test).

For each euthanization time point, blood glucose levels were compared between the STZ group and the sham group at each corresponding time point in the glucose tolerance test using a two‐tailed *t*‐test. Surprisingly, no significant differences were observed between the STZ and sham groups on days 22 and 35 (Figure 4A,B). However, on day 84, the STZ group showed a trend toward lower blood glucose levels (9.6 ± 1.9 mmol/L) compared to the sham group, although this difference was not statistically significant (Figure 4B).

Two‐way repeated‐measures ANOVA revealed significant differences in blood glucose levels between STZ‐treated groups (Figure 4A,B). Post hoc comparisons using the Tukey–Kramer method showed that fasting glucose levels (0 h) were significantly higher in the day 22 group compared to day 84 (*p* = .018) (Figure 4A). At 9 h post‐glucose, both day 22 and day 35 groups had higher glucose levels than the day 84 group (*p* = .019 and *p* = .028, respectively) (Figure 4B).

A similar analysis in sham groups showed fasting blood glucose differences with higher fasting glucose levels in the day 35 group compared to day 22 and day 84 (*p* = .02 and *p* = .0021, respectively), but no significant variation at later time points, suggesting natural animal variability (Figure 4A).

Since the STZ groups on days 22 and 35 did not differ from their respective sham groups, this indicates that the STZ treatment had no effect and was unable to induce diabetes‐like hyperglycemia in these groups.

### 3.8 The STZ treatment did not affect blood ketone levels in STZ‐treated animals euthanized at day 22 and 35

Blood ketone levels were measured at the endpoint just before glucose injection. No significant differences were found when comparing the STZ groups to their respective sham groups with a two‐tailed *t*‐test (Figure 4C). Surprisingly, both the STZ‐ and sham‐treated groups on day 84 had significantly lower ketone levels than the STZ groups on days 22 and 35 (one‐way ANOVA with Tukey's HSD/Kramer post‐hoc test: *p* = .021 and *p* = .0033, respectively) (Figure 4C). These findings suggest that the STZ treatment did not significantly affect blood ketone levels in these groups.

### 3.9 Lower number of insulin‐positive cells in STZ‐treated animals on day 84, but not day 22 or 35

Insulin‐positive cells were quantified using immunofluorescence staining to assess the impact of STZ on pancreatic β cell mass (Figure 4D,E). There was no significant difference in the percentage of insulin‐positive cells between STZ‐treated and sham‐treated animals on days 22 and 35 (two‐tailed *t*‐test, *p* = .20 and *p* = .99, respectively) (Figure 4D), supporting the glucose tolerance test results that these animals did not develop hyperglycemia or diabetes.

However, on day 84, the STZ group showed a significantly lower percentage of insulin‐positive cells (1.82% ± 0.73%) compared to the sham group (3.97% ± 0.73%) (two‐tailed *t*‐test: *p* = .022) (Figure 4D), indicating reduced β cell mass due to STZ treatment. This reduction was unexpected as blood glucose levels had normalized at this time point. This could indicate that, though the β cell mass was not replenished by day 84, the remaining insulin‐positive cells were sufficient for glucose control.

### 3.10 Cell proliferation was unchanged on day 35

To investigate if the axolotl regenerates β cells after the STZ treatment and if a proliferative response is the cause of the blood glucose normalization seen on day 84, the pancreas of the STZ group and the sham group on day 35 were stained for proliferative cells using an EdU kit to detect DNA proliferation (Figure 4F,G).

There was no significant difference in the percentage of proliferating cells between the STZ group and the sham group (two‐tailed *t*‐test: *p* = .20) (Figure 4F), and proliferation was scattered in the tissue, not centered in the endocrine islets (Figure 4G).

### 3.11 STZ treatment induced weight gain and edema in some animals

**FIGURE 5:**
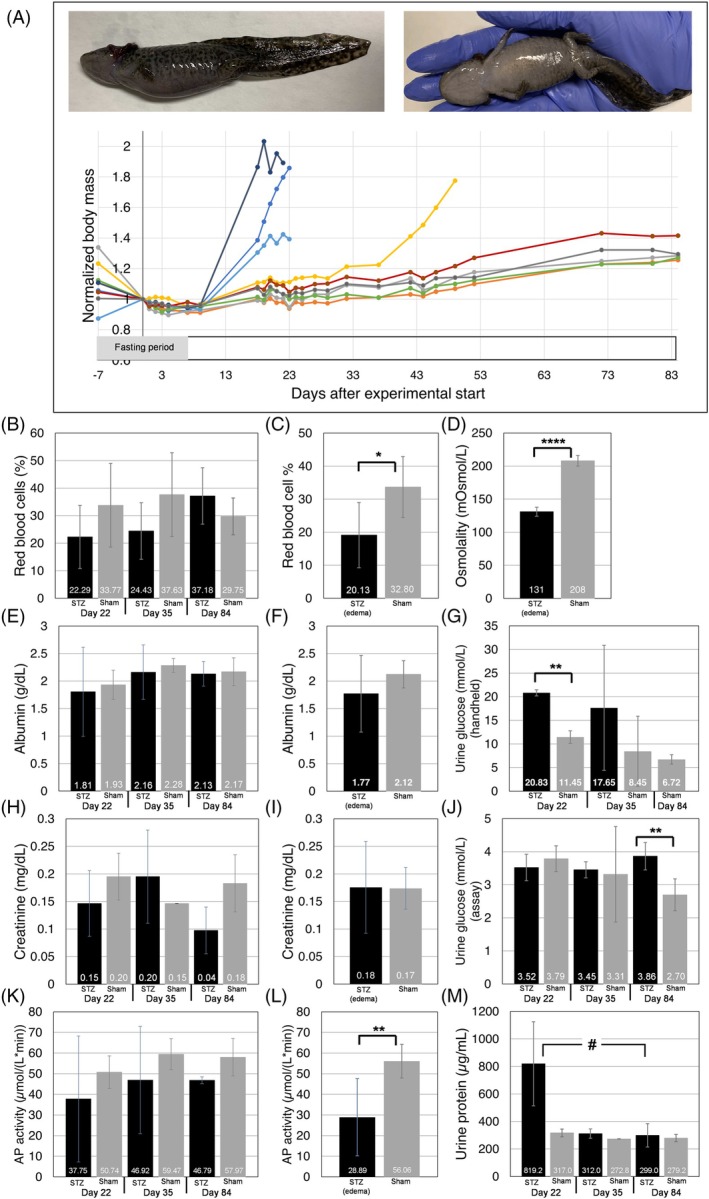
Systemic effects of streptozotocin (STZ) treatment in axolotls. (A) STZ‐induced edema led to severe swelling, increasing body mass by 40%–100% and requiring euthanasia. Individual body mass was normalized to start weight. (B) Red blood cell composition remained unchanged in STZ‐treated animals (day 22: *n* = 6; day 35: *n* = 3; day 84: *n* = 5) compared to sham‐treated animals (day 22: *n* = 4; day 35: *n* = 3; day 84: *n* = 6), but those with edema had lower red blood cell percentages (C) and reduced blood osmolarity (D). Blood albumin (E, F), creatinine (H), and alkaline phosphatase (AP) activity (K, L) were unaffected across groups (*n* = 3–4 per group). Urine glucose levels (J) were elevated in STZ‐treated animals compared to sham controls on day 84 (STZ: day 22: *n* = 4; day 35: *n* = 2; day 84: *n* = 5; sham: day 22: *n* = 2; day 35: *n* = 3; day 84: *n* = 6). In contrast, urine protein levels (M) were higher in STZ‐treated animals on day 22 compared to later time points (STZ: day 22: *n* = 3; day 35: *n* = 2; day 84: *n* = 5; sham: day 22: *n* = 2; day 35: *n* = 2; day 84: *n* = 6). *p < .05, **p < .01, ***p < .001, and ****p < .0001 (two‐tailed *t*‐tests); # *p* < .05, ## *p* < .01, ### *p* < .001, and #### *p* < .0001 (ANOVA with Tukey's HSD/Kramer post‐hoc test or Kruskal–Wallis with Dunn's post‐hoc test if sample size *n* < 3).

Even though STZ is known to selectively target β cells, STZ has been seen to cause nonspecific toxicity.^42^ Of the 19 STZ‐treated animals, 10 exhibited weight gain due to edema (Figure 5A). This affected STZ groups euthanized at all time points (day 22: 4/6, day 35: 2/4, and day 84: 4/9). These animals were euthanized and excluded from the analysis.

Weight changes occurred rapidly, mainly around day 20, with one animal showing edema after 43 days. Autopsy confirmed edema as the cause of weight gain, with fluid accumulation in the pericardium, skin, and abdomen, slowing the heart rate. Swollen animals also had punctured bladders.

### 3.12 The osmolarity and red blood cell fraction were abnormal in the blood of the swollen animals

Hyperosmolarity is seen in diabetic patients and STZ‐treated animal models.^43^ To investigate if the STZ treatment influenced the concentration of osmolytes in the animals that suffered from edema, plasma osmolarity was measured (Figure 5D). Mean plasma osmolarity was 131 ± 6.8 mOsmol/L for Pilot 4 and Pilot 5 (*n* = 5), which is significantly lower than the osmolarity of healthy animals, 208 ± 8 mOsmol/L (*n* = 6) (two‐tailed *t*‐test: *p* = 4.17 × 10^−8^) (Figure 5D).

The fraction of red blood cells in whole blood was also assessed, revealing no difference between the STZ groups and their respective sham groups (Figure 5B). However, swollen STZ‐treated animals (*n* = 7) had a significantly lower red blood cell fraction compared to sham‐treated animals (*n* = 13) (two‐tailed *t*‐test: *p* = .011) (Figure 5C). As only swollen animals exhibited hypoosmolarity and a reduced fraction of red blood cells, this must be considered a secondary rather than a direct effect of the STZ treatment.

### 3.13 Blood creatinine, albumin, and alkaline phosphatase activity were not altered in STZ‐treated animals, but animals affected by edema had lowered alkaline phosphatase activity in the blood

STZ has been reported to cause hepatic and renal failure.^44^, ^45^, ^46^ To address this, albumin concentration, creatinine concentration, and alkaline phosphatase activity were measured in plasma to study liver and kidney function. Plasma was analyzed from the same 3–4 animals from each group. There were no differences between the STZ groups and sham groups in albumin (Figure 5E,F), creatinine (Figure 5H,I), or alkaline phosphatase (Figure 5K). However, when comparing pooled data from edema‐affected animals with the sham groups, a significant reduction in alkaline phosphatase activity was observed (two‐tailed *t*‐test: *p* = .0024) (Figure 5L). These findings highlight the systemic impact of edema, warranting further investigation into its underlying causes.

### 3.14 Urine glucose levels were higher in the STZ group on day 84

In diabetic patients, the body regulates hyperglycemia by excreting glucose in the urine when the glucose amount surpasses the amount that can be reabsorbed by the proximal tubules in the kidneys, resulting in glycosuria.^47^ Changes in urine glucose levels can therefore be used to identify diabetes. Therefore, glucose amount was measured in axolotl urine, first using the handheld device used for blood glucose measurements, and secondly by performing a glucose assay (Figure 5E,J). Due to edema‐related bladder punctures, we were not able to collect urine from all animals.

Using the handheld device, urine glucose was 20.8 ± 0.6 mmol/L in the STZ group at day 22, significantly higher than in the sham group (11.45 ± 1.3 mmol/L, two‐tailed *t*‐test: *p* = .0016) (Figure 5G). No significant difference was found on day 35.

Urine glucose levels were similar between STZ‐treated and sham‐treated animals at days 22 and 35; however, due to low sample sizes in the sham group at day 22 and the STZ group at day 35, statistical comparisons could not be performed at these time points (Figure 5J). At day 84, where sample sizes were sufficient, urine glucose was significantly higher in STZ‐treated animals (3.9 ± 0.4 mmol/L) compared to sham controls (2.7 ± 0.5 mmol/L; two‐tailed *t*‐test: *p* = .0021), indicating increased glucose excretion. This could also imply kidney function inhibition by STZ, though blood tests did not indicate renal dysfunction. The glucose assay was found to be more reliable than the handheld device for urine glucose measurements.

### 3.15 Urine protein levels were unaltered in STZ‐treated animals

Diabetes can affect the kidneys and cause diabetic nephropathy, which is characterized by proteinuria.^48^ To investigate this in the axolotl, urine protein levels were measured in animals on day 22 (STZ: *n* = 3, sham: *n* = 2), day 35 (STZ: *n* = 2, sham: *n* = 2), day 84 (STZ: *n* = 5, sham: *n* = 6) (Figure 5M). Two‐tailed *t*‐tests revealed no significant differences between the STZ‐treated groups and their respective sham controls at any time point. Within the STZ‐treated group, a Kruskal–Wallis test followed by Dunn's post hoc comparison revealed a significant decrease in urinary protein levels from day 22 to day 84 (*p* < .015), suggesting an early peak in proteinuria that diminishes over time (Figure 5M).

## 4 DISCUSSION

This study aimed to establish a reliable protocol for STZ‐induced β cell ablation in axolotls, providing a foundation for future investigations into pancreatic β cell regeneration. Since the axolotl has not previously been used in diabetes research, we first optimized an STZ‐induced diabetes protocol. We have previously tested a protocol (0.35 mg/g STZ over the span of 19 days), inspired by a previous study in zebrafish,^40^ but this resulted in severe systemic toxicity and high mortality (unpublished data). Instead, we tested five different treatment regimens inspired by mouse studies, in which the glycemic state of the animals was evaluated using the glucose tolerance test to measure the metabolic activity of glucose. Of the five treatment regimens, two protocols, PS1 (0.05 mg/g STZ for five consecutive days) and PS3 (0.2 mg/g STZ once), caused hyperglycemia, while higher doses led to edema. PS1 was chosen as the most effective protocol, as it rendered the animal significantly hyperglycemic without causing severe toxicity, in line with studies suggesting smaller doses of STZ are less harmful.^42^

The glucose tolerance test was used to confirm hyperglycemia. The test was optimized for axolotls with additional measurements over a 24‐h time span. Although blood glucose varied in the sham group between test days at 10 min post‐injection, the measurements after 14 and 24 h remained consistent, indicating that early fluctuations did not impact overall results. This variation is likely due to uneven glucose distribution in the bloodstream shortly after injection, making the 10‐min measurement an unreliable indicator of true blood glucose levels.

Since accurate glucose injection is crucial for the glucose tolerance test as a diagnostic tool, the variations in body mass due to edema raised the question of whether glucose doses should account for excess fluid. However, the blood glucose levels measured in the longitudinal group showed no significant difference between swollen and non‐swollen animals, suggesting that the assumption of glucose distribution throughout the body is valid.

The test also demonstrated that minimizing blood loss, by using a handheld device instead of an assay requiring large blood samples, and collecting blood at fewer time points, can reduce mortality rates.

In the first longitudinal glucose monitoring study, we observed that STZ‐treated animals were hyperglycemic on day 22, but their blood glucose normalized by day 72. However, fasting blood glucose remained higher in the STZ group during the 72 days, suggesting the animals had not fully recovered. Histological analysis of the pancreas showed a reduction in β cell mass, but by day 84, their fasting glucose levels normalized, indicating recovery of β cell function, despite only 45.7% of the β cell mass being present.

The significant reduction in insulin‐positive cells in STZ‐treated animals on day 84 compared to sham controls supports that STZ successfully induced β cell loss in this model. Interestingly, despite this reduction, the animals regained glycemic control, suggesting potential compensatory mechanisms such as enhanced function of remaining β cells or early stages of regeneration. Identification of insulin‐producing cells was based on immunofluorescence labeling using a monoclonal antibody targeting insulin/proinsulin. While islet‐localized cytoplasmic staining was clearly observed, a nuclear signal was also consistently present. Although such nuclear localization is uncommon, it is not unheard of. It has previously been reported that the insulin‐insulin receptor complex may translocate to the nucleus after activation.^49^, ^50^ Also, the staining pattern was highly specific to pancreatic endocrine regions. Standard antibody validation controls, including omission of primary and secondary antibodies and staining of non‐endocrine tissues (e.g., liver), showed no background signal. In the absence of axolotl knockout models, these approaches provide the best available validation, and we consider the staining a reliable marker of insulin‐positive cells.

We initially attempted to assess apoptotic cell death using a TUNEL assay on pancreatic tissue sections from STZ‐treated axolotls. However, despite following the established protocol, we encountered issues with non‐specific staining across the entire pancreas, which could not be resolved in this study. While we recognize that this limitation is a potential gap in the study, we focused on the reduction of insulin‐positive cells and the observed hyperglycemia as strong indicators of β‐cell damage and death. We acknowledge that direct evidence of apoptosis would have strengthened our conclusions and propose that future studies focus on optimizing this approach or using alternative methods to confirm apoptotic cell death in axolotls.

In addition, we explored the possibility of measuring blood insulin levels to provide further insight into β‐cell function. However, despite efforts to use an available mouse insulin ELISA kit, we encountered challenges due to the low insulin levels in axolotl blood, which were below the detection threshold of the assay. This limitation prevented us from including insulin measurements in this study. Future work should aim to assess alternative assays that can more sensitively measure insulin levels in axolotl plasma to enhance our understanding of β‐cell function and regeneration in this species.

Surprisingly, in the next round of experiments the STZ‐treated animals euthanized on days 22 and 35 did not develop hyperglycemia or showed a reduction in β cell mass. This may be due to biological variation, as these STZ and sham groups came from mixed parentage with varying ages and sizes. In contrast, the pilot groups and the STZ and sham group in the longitudinal study and euthanized on day 84 were all from the same batch (Figure S3). Future studies should aim to minimize these variables by grouping only animals from the same batch whenever possible to achieve more consistent results.

The STZ group that developed hyperglycemia also showed an initial elevation in blood ketone levels on day 22, which decreased over time, similar to what was observed in the glucose measurements. This suggests that the animals may have relied on this as an alternative metabolic fuel when glucose was not available, similar to diabetic humans and mammalian animal models.

Edema was a major issue in this study, with STZ‐treated animals experiencing fluid accumulation throughout the whole body. Even though it is still unknown what caused edema, a method to drain the animals of excessive fluid could be investigated to avoid loss of animals. However, there is a risk that drainage would be a short‐term solution, and that the animals would swell up again shortly after drainage. Another solution could be to house the animals in isosmotic water (Amphibian Ringer's solution), as our group previously has prevented edema in long‐term anesthetic animals by adjusting the osmolarity of the housing water.^51^

A complicated element of using STZ is the high levels of toxicity and dangers to human health. This is perhaps a greater challenge in an aquatic species compared to mammals, as large volumes of STZ‐contaminated water are produced and the risk of splashing droplets contaminated with STZ poses an added risk to researchers.

Since a diabetes‐like state of hyperglycemia was not induced in animals in the day 35 STZ group intended for assessment of β cell proliferation, the regenerative potential of the axolotl pancreas after chemical ablation of β cells with STZ could not be fully assessed. Future studies could potentially refine the STZ protocol and should focus on investigating β cell regeneration after successfully inducing diabetes in more standardized groups consisting of similarly sized animals from the same batch.

While the STZ model in mice is well‐established for studying β‐cell apoptosis and hyperglycemia, the axolotl offers unique advantages due to its robust, intrinsic regenerative capacity. Unlike mice, which require genetic manipulation or specific conditions (e.g., pregnancy) to induce β‐cell regeneration, the axolotl naturally regenerates complex tissues, including its pancreas. This makes it an ideal model for studying spontaneous β‐cell regeneration within a naturally regenerative system, one that captures the full, multi‐step, and systemically integrated process of regeneration, unlike genetically engineered models, which might force a partial regenerative response by targeting individual genes and may miss the broader interplay.

Our study demonstrates that a diabetes‐like state can be induced in axolotl, with hyperglycemia and a reduced number of insulin‐positive cells observed. However, high variation among animals, complications with edema, and inconclusive histological results prevented definitive conclusions on β‐cell regeneration. Despite these challenges, optimizing procedures could make the axolotl a valuable model for studying β‐cell regeneration. It offers the potential to be used in future studies comparing to mouse and zebrafish models to identify sources of new β cells and the pathways underlying regeneration.

## FUNDING INFORMATION

Novo Nordisk Foundation (NNF21OC0071970) and Riisfort Fonden (RF 8 Oct 2021).

## CONFLICT OF INTEREST STATEMENT

The authors declare no conflicts of interest.

## Supporting information


**FIGURE S1:** Animal information. Overview of animal models for studying regeneration and diabetes, highlighting their advantages and limitations. Figure elements adapted from BioRender.com.

**FIGURE S2:** Study design. (A) Timeline of five pilot studies and a previous axolotl study, showing STZ injection schedules over 22 days. (B) Experimental setup for assessing disease progression and potential regeneration in STZ‐treated axolotls. Animals were euthanized at key time points: peak hyperglycemia (day 22), early regeneration (day 35), and restored blood glucose (day 84). Figure elements adapted from BioRender.com.

**FIGURE S3:** Animal information. Data table of all animal groups used in the pilot studies and the main studies, showing body mass, length, color, sample size, and parentage.

## Data Availability

Raw data pertaining to the study is available upon reasonable request.
