# Supplementary figures and images for: Streptozotocin induced hyperglycemia in the axolotl
Source: Dev Dyn. 2025 Jul 23;255(6):567–84. doi: 10.1002/dvdy.70063 (PMC13260882; doi:10.1002/dvdy.70063)

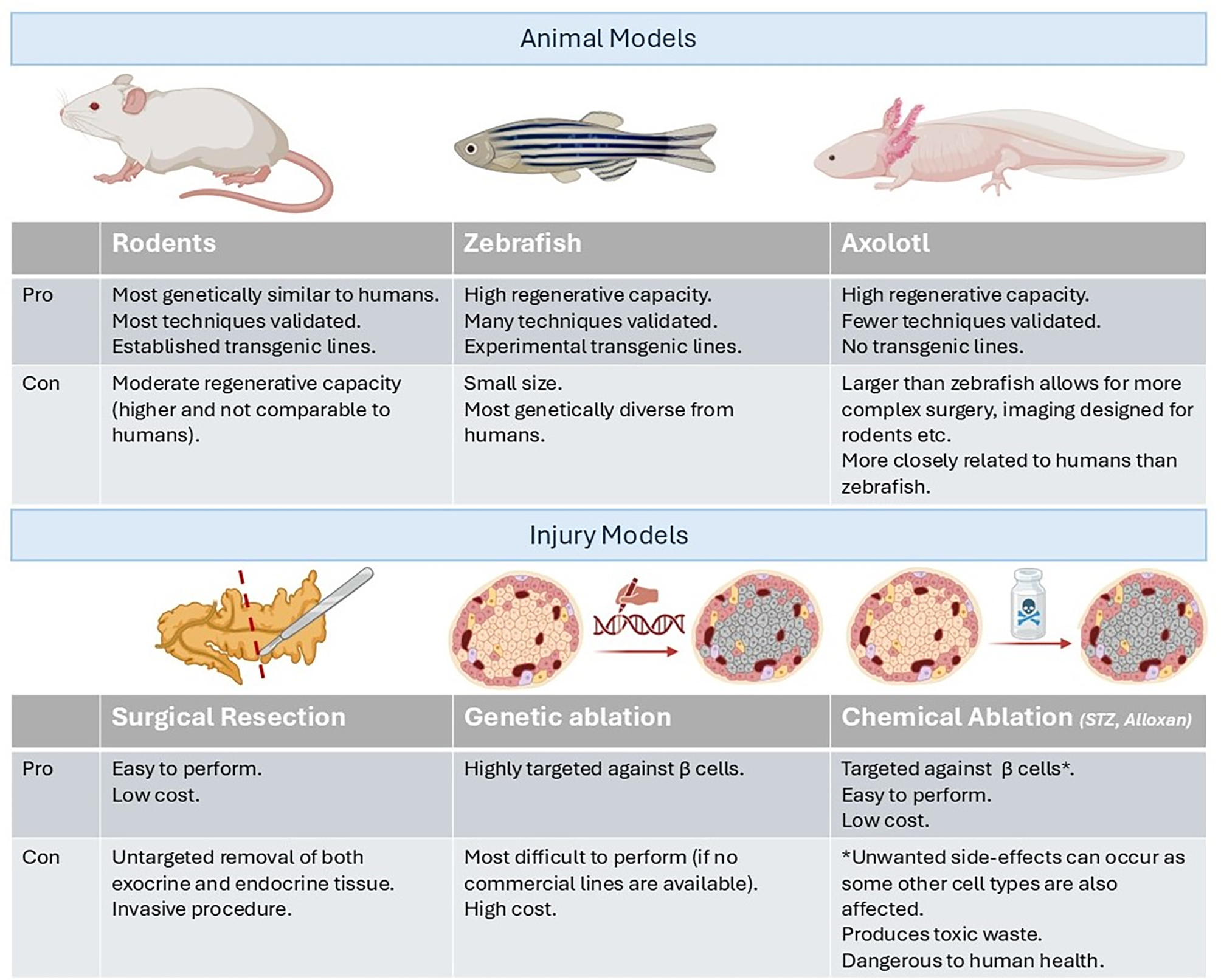

Supplement: Supplementary file 1 — FIGURE S1: Animal information. Overview of animal models for studying regeneration and diabetes, highlighting their advantages and limitations. Figure elements adapted from BioRender.com. [file DVDY-255-567-s002.tif]

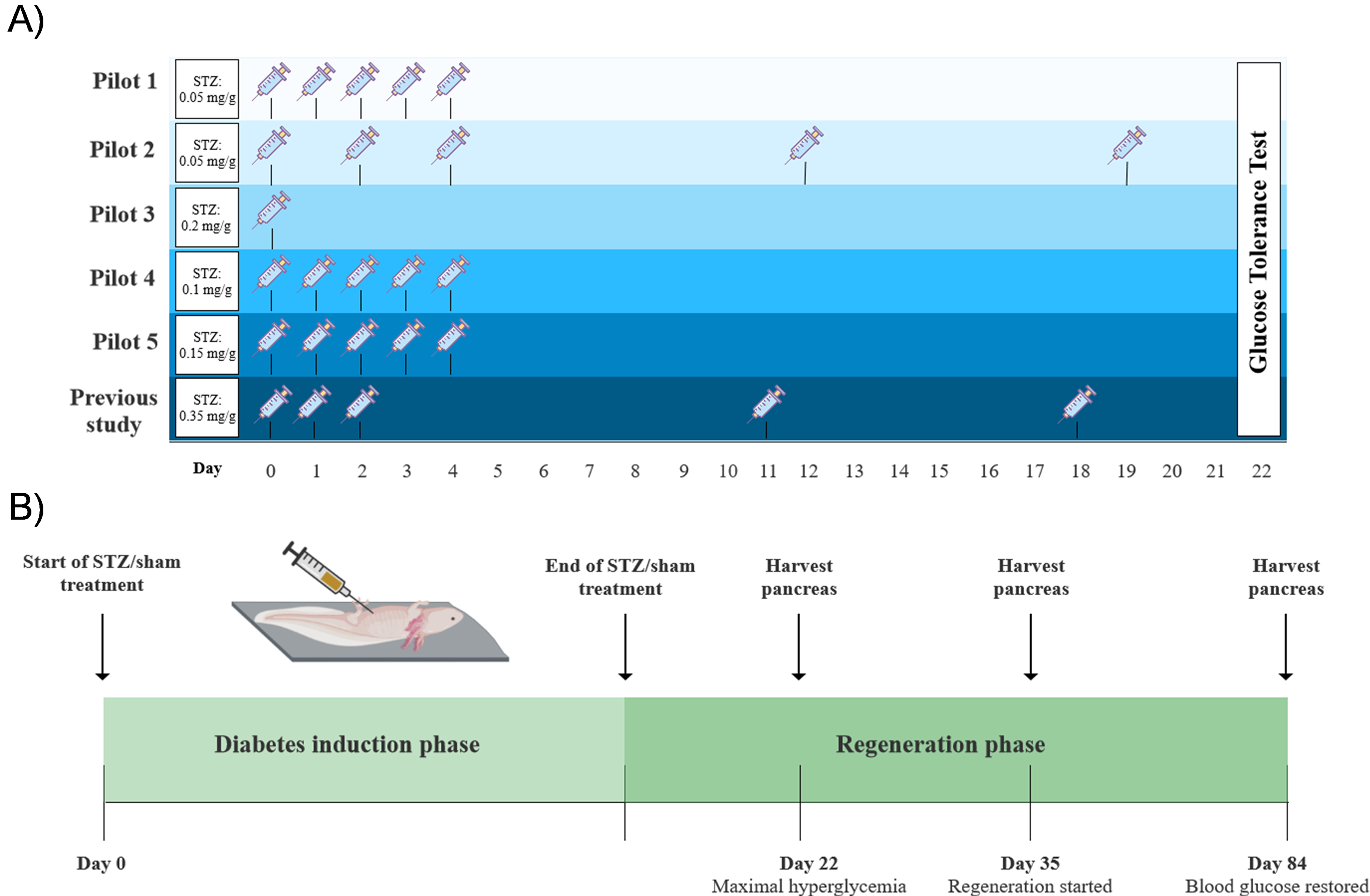

Supplement: Supplementary file 2 — FIGURE S2: Study design. (A) Timeline of five pilot studies and a previous axolotl study, showing STZ injection schedules over 22 days. (B) Experimental setup for assessing disease progression and potential regeneration in STZ‐treated axolotls. Animals were euthanized at key time points: peak hyperglycemia (day 22), early regeneration (day 35), and restored blood glucose (day 84). Figure elements adapted from BioRender.com. [file DVDY-255-567-s003.tif]

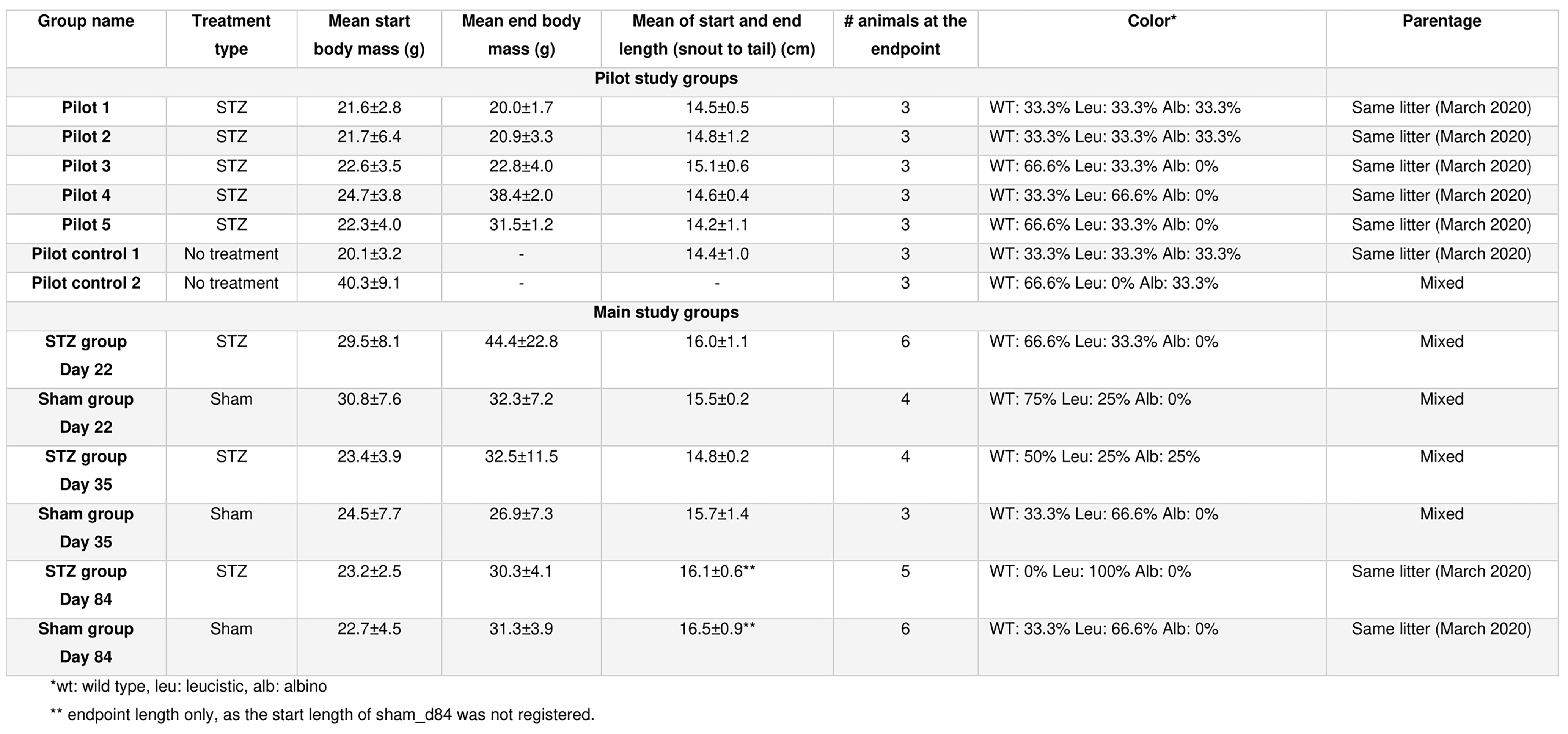

Supplement: Supplementary file 3 — FIGURE S3: Animal information. Data table of all animal groups used in the pilot studies and the main studies, showing body mass, length, color, sample size, and parentage. [file DVDY-255-567-s001.tif]
